# Reply to Jones et al. Comment on “Suresh et al. The Short-Term Effects and Tolerability of Low-Viscosity Soluble Fibre on Gastroparesis Patients: A Pilot Clinical Intervention Study. *Nutrients* 2021, *13*, 4298”

**DOI:** 10.3390/nu14091848

**Published:** 2022-04-28

**Authors:** Harsha Suresh, Jerry Zhou, Vincent Ho

**Affiliations:** 1 School of Medicine, Western Sydney University, Campbelltown, NSW 2560, Australia; 17271790@student.westernsydney.edu.au (H.S.); v.ho@westernsydney.edu.au (V.H.); 2 Gastrointestinal Motility Disorders Unit, Western Sydney University, Campbelltown, NSW 2560, Australia; 3 University Medical Clinic of Camden & Campbelltown (UMCCC), Campbelltown, NSW 2560, Australia

We would like to thank Jones and colleagues for taking the time to comment [1] on our recent study [2]. Their feedback is greatly appreciated, and we will take it into considering in designing our future research.

It is well known that there is a high prevalence of slow-transit constipation in patients with gastroparesis [3]. It may be that the patients with gastroparesis and slow-transit constipation have a more generalised dysmotility, and in fact, we might expect this from conditions such as Ehlers–Danlos syndrome [4] which are affected systemically rather than locally as might be the case, for example, in post-surgical gastroparesis. Certainly from a clinical perspective, we find that many persons with slow-transit constipation can develop secondarily gastroparesis-like symptoms, especially if they are very impacted. However, I do not believe we can say that all cases of gastroparesis are attributable to slow-transit constipation.

Practically addressing slow-transit constipation in gastroparesis patients is sensible. Jones and colleagues address the point that fibre has been controversial as part of dietary management for gastroparesis and concomitant constipation. Fibre avoidance or minimisation might help the gastric transit time in gastroparesis, but this could potentially worsen constipation. Additionally, fibre is an important source of short-chain fatty acids for colonocytes in the caecum.

I think a new research direction moving on from this might be to investigate what specific fibres might be best tolerated in patients with gastroparesis, with an exploration of their other properties (partly shown in our recent study). Once those ‘optimal’ fibres are found, these can be investigated to see if they are relevant for the treatment of slow-transit constipation, with relevant outcome measures to investigate the intestinal transit time, stool weight, and bowel motion frequency.
